# Validation of electronic health record data to identify hospital-associated *Clostridioides difficile* infections for retrospective research

**DOI:** 10.1017/ice.2024.140

**Published:** 2024-12

**Authors:** Michael J. Ray, Kathleen L. Lacanilao, Maela Robyne Lazaro, Luke C. Strnad, Jon P. Furuno, Kelly Royster, Jessina C. McGregor

**Affiliations:** 1 Oregon State University College of Pharmacy, Department of Pharmacy Practice, Portland, OR, USA; 2 Oregon Health & Science University-Portland State University School of Public Health, Portland, OR, USA; 3 Oregon Health & Science University School of Medicine, Division of Infectious Diseases, Portland, OR, USA; 4 Legacy Health, Pharmacy, Portland, OR, USA

## Abstract

*Clostridioides difficile* infection (CDI) research relies upon accurate identification of cases when using electronic health record (EHR) data. We developed and validated a multi-component algorithm to identify hospital-associated CDI using EHR data and determined that the tandem of CDI-specific treatment and laboratory testing has 97% accuracy in identifying HA-CDI cases.

## Background

Healthcare-associated *Clostridioides difficile* infection (HA-CDI) represents about two-thirds of CDI cases in the United States.^1,2^ Retrospective epidemiologic studies have focused on identifying risk factors, evaluating diagnosis and treatment appropriateness, and measuring attributable outcomes for HA-CDI.^3,4^ However, the validity of this research relies on accurate identification of HA-CDI cases, and previous studies of other infections have demonstrated that reliance on administrative or laboratory data may lead to misclassification.^5,6^

We developed and validated a CDI case definition to accurately detect CDI cases using antibiotic treatment, laboratory test, and diagnosis code data in the electronic health record (EHR) to specifically be used for retrospective research. We hypothesized that a multi-component case definition would more accurately detect patients with CDI compared to any single-component case definition.

## Method

### Study design and data source

This validation study was conducted including all Oregon Health & Science University (OHSU) inpatient hospital encounters between January 2018 and March 2020. OHSU is a 576-bed academic, quaternary-care hospital in Portland, Oregon. We excluded patients under age 18, those with known recurrent or community-acquired CDI, and those with hospital stays of less than four calendar days. Eligible subjects were sampled for chart review as described below and in Supplemental Figure 1. This project was approved by OHSU’s institutional review board.

### Case definition algorithm to identify incident hospital-associated CDI

EHR data were collected from our institution’s previously validated research data repository.^7^ To identify putative cases of incident, non-recurrent HA-CDI, we combined medication, diagnosis code, and laboratory testing data (Box 1). We defined hospital-associated CDI as incident CDI when the onset date, defined as the date of first anti-*C. difficile* antibiotic administration or *C. difficile* positive stool specimen, whichever occurred first, fell on hospital day 4 or later. We defined CDI as non-recurrent if no prior CDI events were identified at our institution in the 8 weeks before the index CDI diagnosis applying the same diagnostic criteria.

Box 1. Case definition for incident hospital-associated *C. difficile* infection cases

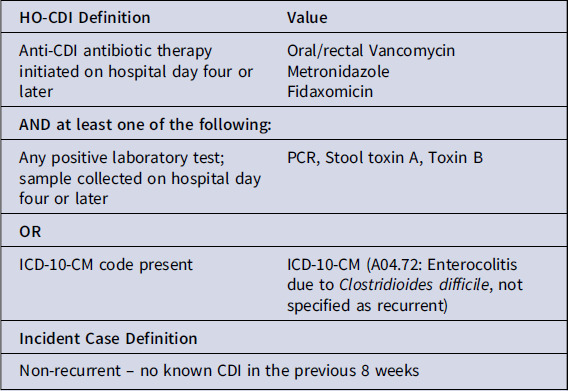

### Collection of gold-standard incident HA-CDI data

We randomly selected 80 algorithm-identified HA-CDI cases and 80 non-CDI cases for chart review to identify the gold-standard “true” case status. We determined *a priori* that this sample size would be sufficient to achieve 94% power to discern cases from non-cases.^8^ We (MJR, KLL, MRL, KR) manually reviewed each encounter medical record (Epic EHR system). To be ruled a true case of HA-CDI and establish our gold standard, there must have been documentation of, on hospital day 4 or later, at least three loose/liquid/unformed stools with no alternative explanation documented for diarrhea symptoms, initiation of CDI-specific antibiotic treatment, and any positive laboratory test (*C. difficile* toxin or PCR if toxin test indeterminant) for *C. difficile* or *C. difficile*-specific diagnosis code. We flagged the record for further review by an infectious disease physician (LCS) or pharmacist (KR) if the initial reviewer was unable to reach a CDI ruling. We utilized REDCap to collect study data.

### Data analysis

We calculated our case algorithm’s sensitivity, specificity, positive predictive value (PPV), negative predictive value (NPV), and overall percent accuracy (ie, percent of cases/non-cases correctly identified) with 95% confidence intervals (CI) for each.^8^ Chart review assessment was considered the gold standard and case identification based on electronic data were considered “test” data. We also examined the diagnostic performance of individual algorithm components (eg, laboratory test only, diagnosis code only, oral vancomycin only) and various modifications to the algorithm (Box 1).Our power calculation was performed using Stata (version 16, StataCorp., College Station, TX) and all other analysis using SAS (v9.4, SAS Corporation, Cary, NC).

## Results

Of the 103,275 inpatient encounters evaluated, 50,394 (49%) were eligible for inclusion. Overall, 5,039 (10%) of included encounters involved CDI treatment (metronidazole, oral vancomycin, or fidaxomicin), with 710 (14.1%) receiving oral vancomycin or fidaxomicin. A positive test for *C. difficile* was identified in 396 encounters (0.8%), and 487 (1%) had an ICD-10 code for non-recurrent CDI. Per our case definition (Box 1), we identified 190 putative cases of incident, HA-CDI. Among these, 157 (83%) encounters had all three components of our case definition (anti-CDI therapy, positive laboratory test, and ICD-10 code). Of the 80 algorithm-identified HA-CDI cases that we sampled for review, 66 (83%) had all three criteria, while 9 (11%) had a positive laboratory test and no ICD-10 code, and 5 (6%) had an ICD-10 code and no positive laboratory test.

Among our chart review sample, our algorithm identified HA-CDI cases with 94% accuracy (95% CI: 88%–97%). We achieved 100% sensitivity (94%–100%), 89% specificity (81%–95%), 88% PPV (78%–94%), and 100% NPV (95%–100%). Performance of the individual algorithm components is summarized in Table 1. Adapting the initial algorithm to require a positive laboratory test (as opposed to an optional positive test if an ICD-10 code for CDI was included) improved diagnostic performance across all measures by avoiding 5 false positives, compared to the original algorithm, improving specificity to 94% (87%–98%), PPV to 93% (84% –98%), and overall accuracy to 97% (93%–99%).

**Table 1.** Diagnostic performance of our CDI algorithm and comparison of individual algorithm components among 80 algorithm-identified HA-CDI cases and 80 non-cases

Case definition criteria	Diagnostic performance measure, % (95% CI)	
**Initial study algorithm:** *ICD-10-CM code A04.72 OR positive C. difficile laboratory test AND CDI-specific antibiotic (metronidazole, oral vancomycin, fidaxomicin)*	Sensitivity	100 (94–100)
	Specificity	89 (81–95)
	PPV	88 (78–94)
	NPV	100 (95–100)
	Accuracy	94 (88–97)
**Revised study algorithm:** *Positive C. difficile laboratory test AND CDI-specific antibiotic*	Sensitivity	100 (94–100)
	Specificity	94 (87–98)
	PPV	93 (85–98)
	NPV	100 (96–100)
	Accuracy	97 (93–99)
**Code required:** *ICD-10-CM code A04.72 AND CDI-specific antibiotic*	Sensitivity	93 (84–98)
	Specificity	94 (87–98)
	PPV	93 (84–98)
	NPV	94 (87–98)
	Accuracy	94 (88–97)
**Any CDI drug only:** *CDI-specific antibiotic (metronidazole, oral vancomycin, fidaxomicin)*	Sensitivity	74 (64–83)
	Specificity	73 (63–82)
	PPV	74 (64–83)
	NPV	100 (95–100)
	Accuracy	85 (78–90)
**Oral vancomycin only**	Sensitivity	100 (94–100)
	Specificity	79 (69–87)
	PPV	79 (68–87)
	NPV	100 (94–100)
	Accuracy	88 (82–93)
**Positive** ***C. difficile*** **laboratory test only**	Sensitivity	100 (94–100)
	Specificity	83 (74–90)
	PPV	82 (72–90)
	NPV	100 (95–100)
	Accuracy	91 (85–95)

Abbreviations: CI, confidence interval; PPV, positive predictive value; NPV, negative predictive value.

## Discussion

Our study suggests that the best strategy to identify inpatient HA-CDI cases relies on a combination of drug administration and laboratory testing test; use of ICD-10 code data does not improve case identification. Requiring a positive *C. difficile* laboratory test further improved the diagnostic accuracy by avoiding 5 false positives, and use of multiple components performed better than any individual component.

Our study advances the methodological foundation for future retrospective epidemiologic studies of HA-CDI by providing a validated, accurate method for case identification. Much of the literature to date examines the utility of using a single component to detect cases. For example, Litvin et al. observed a “pseudo-outbreak” of CDI using a laboratory-test-only-based definition, which was, in reality, due to a faulty assay lot leading to a perceived 32% facilitywide increase in CDI incidence.^9^ Pfister et al. reported that the ICD-10-CM code for non-recurrent CDI had 85% sensitivity and 80% PPV when applied to a provincewide (Alberta, CA) discharge database.^10^ These studies identify important pitfalls of single-component case detection, thus motivating our study.

The primary limitation to this study is the assessment of the gold-standard HA-CDI diagnosis, which relies on EHR documentation and may not align with a prospective case evaluation, had that been feasible. Further assessment at additional facilities is necessary to determine if these results are generalizable, given differences in patient acuity, CDI incidence, testing, and antibiotic utilization. Additionally, while we calculated power/sample size *a priori*, it is possible that we underestimated our denominator for sensitivity and NPV calculations, though this would not affect our specificity and PPV calculations. Finally, we are unable to elucidate if an individual had CDI at another institution. Thus, we could be misclassifying recurrent CDI as initial episodes.

Our study has important implications. Our CDI case definition algorithm can be applied as a gold standard to readily available EHR information to accurately detect HA-CDI cases. Accurate retrospective identification of CDI cases is crucial for research as misclassification could lead to biased estimates of risk. Our algorithm detected HA-CDI cases with perfect sensitivity and high overall accuracy. Requiring a positive laboratory test further improved our algorithm’s diagnostic accuracy. We recommend considering both a CDI-specific medication and a positive laboratory test as the new standard research definition when classifying HA-CDI cases from EHR data.

## Supporting information

Ray et al. supplementary material Ray et al. supplementary material

## Supplementary material

The supplementary material for this article can be found at https://doi.org/10.1017/ice.2024.140

## References

[1] US Centers for Disease Control and Prevention. Antibiotic resistance threats in the United States, 2019. Centres for Disease Control and Prevention, US Department of Health and …; 2019.

[2] Lessa FC , Mu Y , Bamberg WM , et al. Burden of *Clostridium difficile* infection in the United States. N Engl J Med 2015;372:825–834. 25714160 10.1056/NEJMoa1408913 PMC10966662

[3] Kang M , Abeles SR , El-Kareh R , et al. The effect of *Clostridioides difficile* diagnostic stewardship interventions on the diagnosis of hospital-onset *Clostridioides difficile* infections. Jt Comm J Qual Patient Saf 2020;46:457–463. 32576438 10.1016/j.jcjq.2020.05.004

[4] Kelly SG , Yarrington M , Zembower TR , et al. Inappropriate *Clostridium difficile* testing and consequent overtreatment and inaccurate publicly reported metrics. Infect Control Hosp Epidemiol 2016;37:1395–1400. 27666285 10.1017/ice.2016.210

[5] Longtin Y , Trottier S , Brochu G , et al. Impact of the type of diagnostic assay on *Clostridium difficile* infection and complication rates in a mandatory reporting program. Clin Infect Dis 2013;56:67–73. 23011147 10.1093/cid/cis840

[6] Marra AR , Edmond MB , Ford BA , Herwaldt LA , Algwizani AR , Diekema DJ. Failure of risk-adjustment by test method for *C. difficile* laboratory-identified event reporting. Infection Control Hosp Epidemiol 2017;38:109–111. 10.1017/ice.2016.227 27745553

[7] Furuno JP , Tallman GB , Noble BN , et al. Clinical outcomes of oral suspension versus delayed-release tablet formulations of posaconazole for prophylaxis of invasive fungal infections. *Antimicrob Agents Chemother* 2018;62:e00893–00818. 10.1128/AAC.00893-18 PMC6153813 30012757

[8] Pepe MS. The Statistical Evaluation of Medical Tests for Classification and Prediction. Oxford: Oxford university press; 2003.

[9] Litvin M , Reske KA , Mayfield J , et al. Identification of a pseudo-outbreak of Clostridium difficile infection (CDI) and the effect of repeated testing, sensitivity, and specificity on perceived prevalence of CDI. Infect Control Hosp Epidemiol 2009;30:1166–1171. 19848606 10.1086/648089 PMC3598603

[10] Pfister T , Rennert-May E , Ellison J , Bush K , Leal J. Clostridioides difficile infections in Alberta: the validity of administrative data using ICD-10 diagnostic codes for CDI surveillance versus clinical infection surveillance. Am J Infect Control 2020;48:1431–1436. 32810568 10.1016/j.ajic.2020.08.016

