# Supplementary material for: Validation of electronic health record data to identify hospital-associated Clostridioides difficile infections for retrospective research
Source: Infect Control Hosp Epidemiol. 2024 Oct 16;45(12):1472–4. doi: 10.1017/ice.2024.140 (PMC11663474; doi:10.1017/ice.2024.140)

**Supplementary material for “Validation of electronic health record data to identify hospital-associated *Clostridioides difficile* infections"**

**Power and sample size calculation**

Methods outlined by Pepe and Longton

<https://research.fredhutch.org/diagnostic-biomarkers-center/en/software.html>

Assumptions:

True positive rate (TPR): 90%

False positive rate (FPR): 5%

Alternate true positive rate: 75%

Alternate false positive rate: 25%

Output from Stata v16:


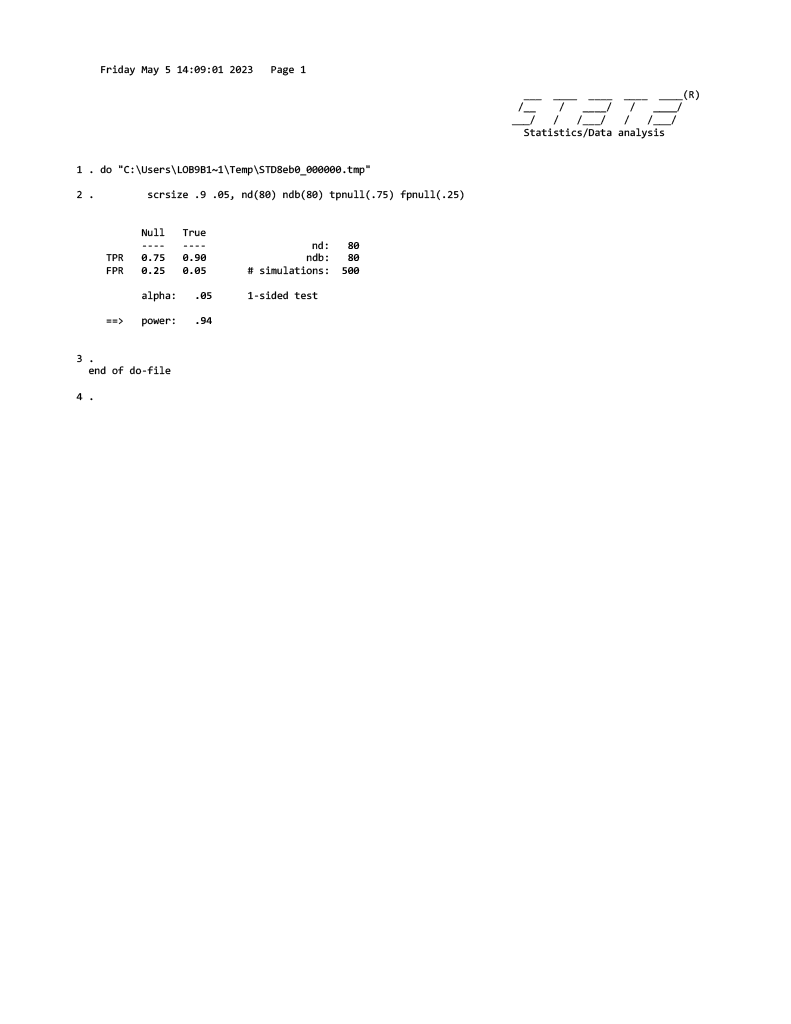


**Supplemental Figure 1.** Inclusion/exclusion criteria and sampling scheme for our validation study


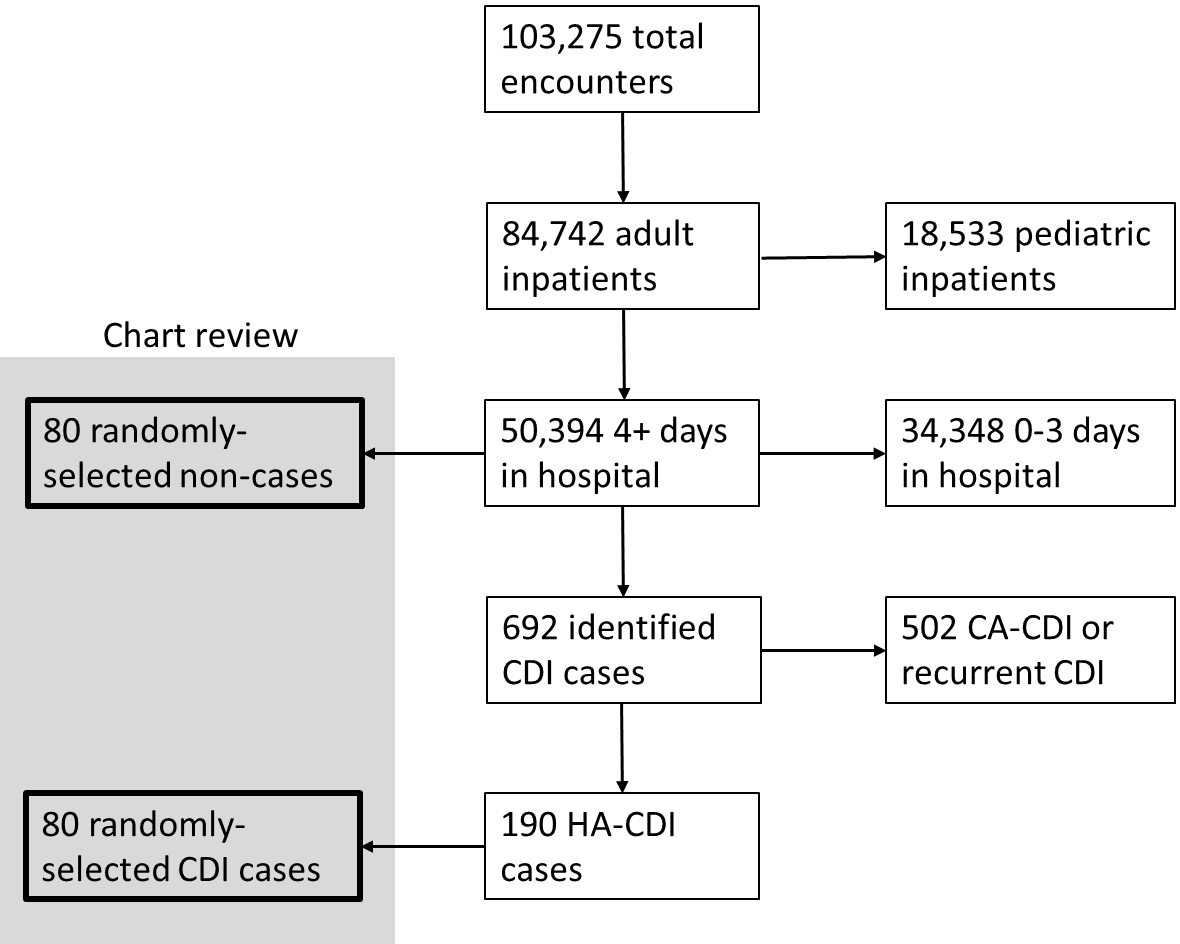

Supplement: Ray et al. supplementary material [file S0899823X24001405sup001.docx]
